# The contribution of cardiopulmonary exercise testing in the familial screening for dilated and non-dilated left ventricular cardiomyopathies: case series

**DOI:** 10.1093/ehjcr/ytaf162

**Published:** 2025-04-08

**Authors:** Eva Del Mestre, Teresa Maria Capovilla, Alessia Paldino, Marco Cittar, Martina Setti, Matteo Dal Ferro, Marco Merlo, Gianfranco Sinagra

**Affiliations:** Center for Diagnosis and Treatment of Cardiomyopathies, Cardiovascular Department, Azienda Sanitaria Universitaria Giuliano-Isontina (ASUGI), University of Trieste, Via Valdoni 7, 34149 Trieste, Italy; European Reference Network for Rare, Low Prevalence and Complex Diseases of the Heart (ERN GUARD—Heart); Center for Diagnosis and Treatment of Cardiomyopathies, Cardiovascular Department, Azienda Sanitaria Universitaria Giuliano-Isontina (ASUGI), University of Trieste, Via Valdoni 7, 34149 Trieste, Italy; European Reference Network for Rare, Low Prevalence and Complex Diseases of the Heart (ERN GUARD—Heart); Center for Diagnosis and Treatment of Cardiomyopathies, Cardiovascular Department, Azienda Sanitaria Universitaria Giuliano-Isontina (ASUGI), University of Trieste, Via Valdoni 7, 34149 Trieste, Italy; European Reference Network for Rare, Low Prevalence and Complex Diseases of the Heart (ERN GUARD—Heart); Cardiology and Cardiothoracic Department, University Hospital ‘Santa Maria della Misericordia’ (ASUFC), Piazzale Santa Maria della Misericordia 15, 33100 Udine, Italy; Division of Cardiology, Department of Medicine, University of Verona, Piazzale Aristide Stefani 1, 37126 Verona, Italy; Center for Diagnosis and Treatment of Cardiomyopathies, Cardiovascular Department, Azienda Sanitaria Universitaria Giuliano-Isontina (ASUGI), University of Trieste, Via Valdoni 7, 34149 Trieste, Italy; European Reference Network for Rare, Low Prevalence and Complex Diseases of the Heart (ERN GUARD—Heart); Center for Diagnosis and Treatment of Cardiomyopathies, Cardiovascular Department, Azienda Sanitaria Universitaria Giuliano-Isontina (ASUGI), University of Trieste, Via Valdoni 7, 34149 Trieste, Italy; European Reference Network for Rare, Low Prevalence and Complex Diseases of the Heart (ERN GUARD—Heart); Center for Diagnosis and Treatment of Cardiomyopathies, Cardiovascular Department, Azienda Sanitaria Universitaria Giuliano-Isontina (ASUGI), University of Trieste, Via Valdoni 7, 34149 Trieste, Italy; European Reference Network for Rare, Low Prevalence and Complex Diseases of the Heart (ERN GUARD—Heart)

**Keywords:** Family screening, Dilated cardiomyopathy, Non-dilated left ventricular cardiomyopathy, Genetic testing, Cardiopulmonary exercise testing, Case series

## Abstract

**Background:**

The importance of family screening in relatives of patients affected by cardiomyopathies is highlighted in the international guidelines. Although electrocardiogram (ECG) and echocardiogram represent cornerstones of family screening, they may not always be sufficient to detect subtle abnormalities, especially in genotype-positive/phenotype-negative relatives. The role of cardiopulmonary exercise testing (CPET) in providing additional clinical information during family screening, remains to be fully elucidated.

**Case summary:**

Ten asymptomatic genotype-positive/phenotype-negative first-degree relatives of probands affected by dilated cardiomyopathy (DCM) and non-dilated left ventricular cardiomyopathy (NDLVC) were evaluated in the context of family screening. Cardiopulmonary exercise testing was integrated into the initial diagnostic evaluation. Two out of 10 relatives showed an abnormal CPET, with alteration in O_2_ kinetic.

**Discussion:**

Family screening in relatives of DCM and NDLVC patients, particularly in genotype-positive/phenotype-negative subjects, remains challenging due to difficulties in assessing the subtle abnormalities that may represent an initial clinical manifestation of the disease and support early treatment initiation. A more accurate and comprehensive familial screening may be achieved by integrating ECG and echocardiogram—the current first-line assessments—with data from additional tools, such as global longitudinal strain on echocardiogram, cardiac magnetic resonance, Holter ECG, and CPET.

**Learning points**

- Due to the heterogeneity of phenotype expression and the incomplete penetrance of dilated cardiomyopathy (DCM) and non-dilated left ventricular cardiomyopathy (NDLVC), family screening in genotype-positive/phenotype-negative relatives should be multiparametric, incorporating family history and electrocardiogram (ECG), echocardiogram, cardiac magnetic resonance, and Holter ECG.
- Cardiopulmonary exercise testing (CPET) may contribute to the evaluation of asymptomatic genotype-positive relatives of patients with DCM or NDLVC, who have a normal left ventricle ejection fraction. Indeed, CPET can identify subtle functional abnormalities in myocardial function, thereby enabling diagnosis and prognostic stratification.

## Introduction

Individuals with a family history of dilated cardiomyopathy (DCM) or non-dilated left ventricular cardiomyopathy (NDLVC) are predisposed to developing these conditions.^1^ This risk is significantly elevated when a pathogenic (P) or likely pathogenic (LP) genetic mutation is identified in the probands and subsequently confirmed in the individual relative. Family screening offers a valuable opportunity to identify these at-risk individuals, who may still be asymptomatic or appear unaffected (genotype-positive and phenotype-negative) at the time of their first medical contact.^2^

The electrocardiogram (ECG), together with echocardiographic evaluation, represents the cornerstone of family screening.^2^ This initial evaluation can identify relatives with overt abnormalities who may receive a diagnosis, enabling monitoring for disease progression, including cardiac events.^3^ However, ECG and echocardiogram may not detect subtle abnormalities.

In addition to the left ventricular ejection fraction (LVEF), which is frequently found to be normal in the majority of relatives at baseline evaluation, the incorporation of other tools, such as assessment of subtle LV dysfunction with global longitudinal strain (LV-GLS) and tissue characterization by the cardiac magnetic resonance (CMR), contributes to a more precise and accurate clinical evaluation.^2,4–6^

Cardiopulmonary exercise testing (CPET) is a comprehensive assessment tool for patients with heart failure (HF). It provides information regarding the patient’s functional capacity, as well as their cardiac, ventilatory, and metabolic response to physical exercise, thus enabling prognostic stratification.^7–9^ The role of CPET in the assessment of family members during screening and evaluation of subtle functional abnormalities remains relatively under-explored. An altered functional capacity and a non-physiological cardiopulmonary response to exercise have been identified as an early indicator of the potential presence of cardiomyopathy that can precede the manifestation of structural alterations at rest.

We included the CPET, used to assess functional capacity (for the CPET protocol used, see Supplementary material online), in the screening of 10 asymptomatic genotype-positive and phenotype-negative (defined as the presence of a normal LVEF) relatives of patients with DCM or NDLVC (*Table 1*). The present Case Series focuses on two out of the 10 screened patients, in whom no abnormalities were detected at the first-line examinations or only minor non-diagnostic abnormalities were identified, illustrating CPET’s potentially informative role in conjunction with other diagnostic methods.

**Table 1:** Summary of the baseline characteristic of the 10 genotype positive/phenotype-negative relatives of probands affected by dilated cardiomyopathy or non-dilated left ventricular cardiomyopathy, screened with the cardiopulmonary exercise testing

	Relative 1	Relative 2	Relative 3	Relative 4	Relative 5	Relative 6	Relative 7	Relative 8	Relative 9	Relative 10
Age	37	39	38	36	59	30	27	52	30	19
Gender	F	F	M	F	M	M	M	F	M	F
Familial cardiomyopathy	DCM	DCM	NDLVC	DCM	NDLVC	DCM	DCM	DCM	DCM	NDLVC
Genetic testing P/LP	TTN	TTN	FLNC	FLNC	FLNC	TTN	TTN	TTN	LMNA	ACTC1
ECG abnormalities*	No	Yes	No	No	No	Yes	No	Yes	No	Yes
Holter ECG abnormalities**	No	Yes	No	No	No	No	No	Yes	No	No
LVEDD (mm)	50	41	46	51	48	57	54	48	49	42
LVEF (%)	57	55	60	56	54	52	61	60	63	63
LV-GLS (%)	−17	−18	−21	−20	−19	−17	−24	−19	−20	−21
LGE	No	/	No	Yes	No	Yes	Yes	Yes	No	No
Peak VO_2_ (mL/kg/min) (% pred.)***	29 (102%)	27 (80%)	33 (93%)	24 (76%)	30 (102%)	45 (111%)	43 (99%)	27 (106%)	31 (81%)	37 (111%)
VO_2_/work (mL/W/min)	8.9	8.8	9.7	8.8	11.3	10.1	10.8	9.7	9.1	10.3
O_2_ pulse (% pred.)	102	83	115	82	113	112	110	109	88	124
VE/VCO_2_ slope	27	31	31	25	25	19	27	32	20	28
Arrhythmias during CPET	No	No	Yes	Yes	Yes	No	No	No	No	No

ACTC1, actin alpha cardiac muscle 1; CPET, cardiopulmonary exertion testing; DCM, dilated cardiomyopathy; F, female; FLNC, filamin C; G+, genotype positive; LGE, late gadolinium enhancement; LMNA, lamin A; LP, likely pathogenetic; LVEDD, left ventricle end diastolic diameter; LVEF, left ventricle ejection fraction (echo); LVGLS, left ventricle global longitudinal strain (echo); M, male; NDLVC, non-dilated left ventricle cardiomyopathy; P, pathogenetic; P−, phenotype negative; TTN, titin; "/": data not available.

^*^ECG abnormalities: Q waves, negative T waves, fragmented QRS complexes, low voltage QRS, and left bundle branch block.^9,10^

^**^Holter ECG abnormalities: VPBs > 500/24 h, non-sustained ventricular tachycardia >3 beats with FC > 150 b.p.m., AVB II or III degree, paroxysmal AF.^1,11^

^***^All CPETs were performed at maximal intensity, with a RER exceeding 1.10.

## Summary figure

**Figure:**
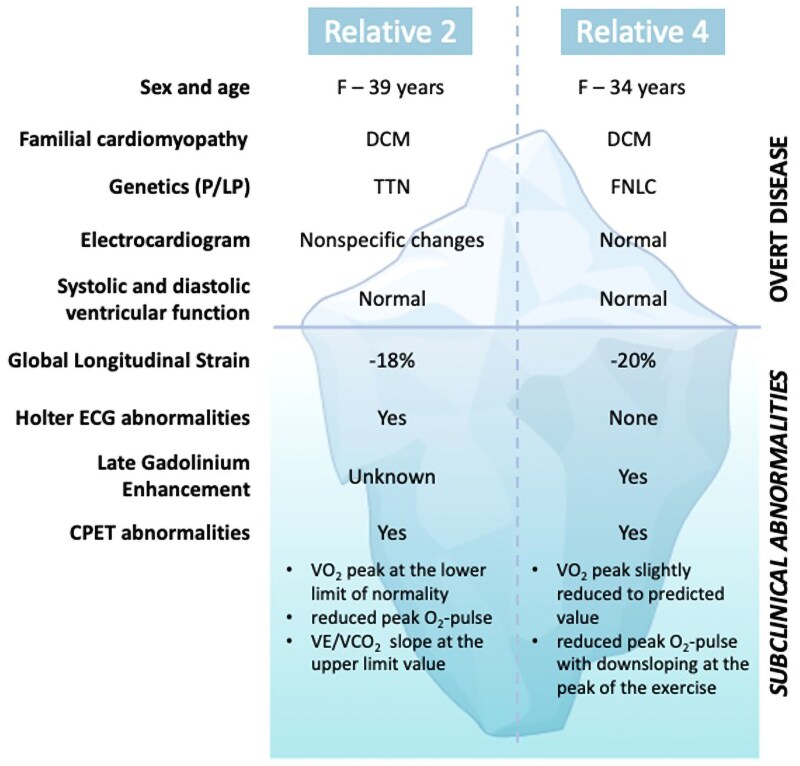


## Relative 2: titin

A 39-year-old female was examined for the first time in the context of family screening. Her father (the proband) was diagnosed as having DCM caused by a heterozygous LP variant within the Titin (*TTN*) gene (NM_001267550.2: c.11389del; p.Thr3797Hisfs*35). Genetic testing showed the patient has the same heterozygous variant.

Family history revealed no instances of sudden cardiac death or life-threatening arrhythmias. The daughter had no relevant medical history. She engages in regular, non-intensive, non-competitive physical activity. A single pregnancy was carried to term without complications.

**Figure 1:**
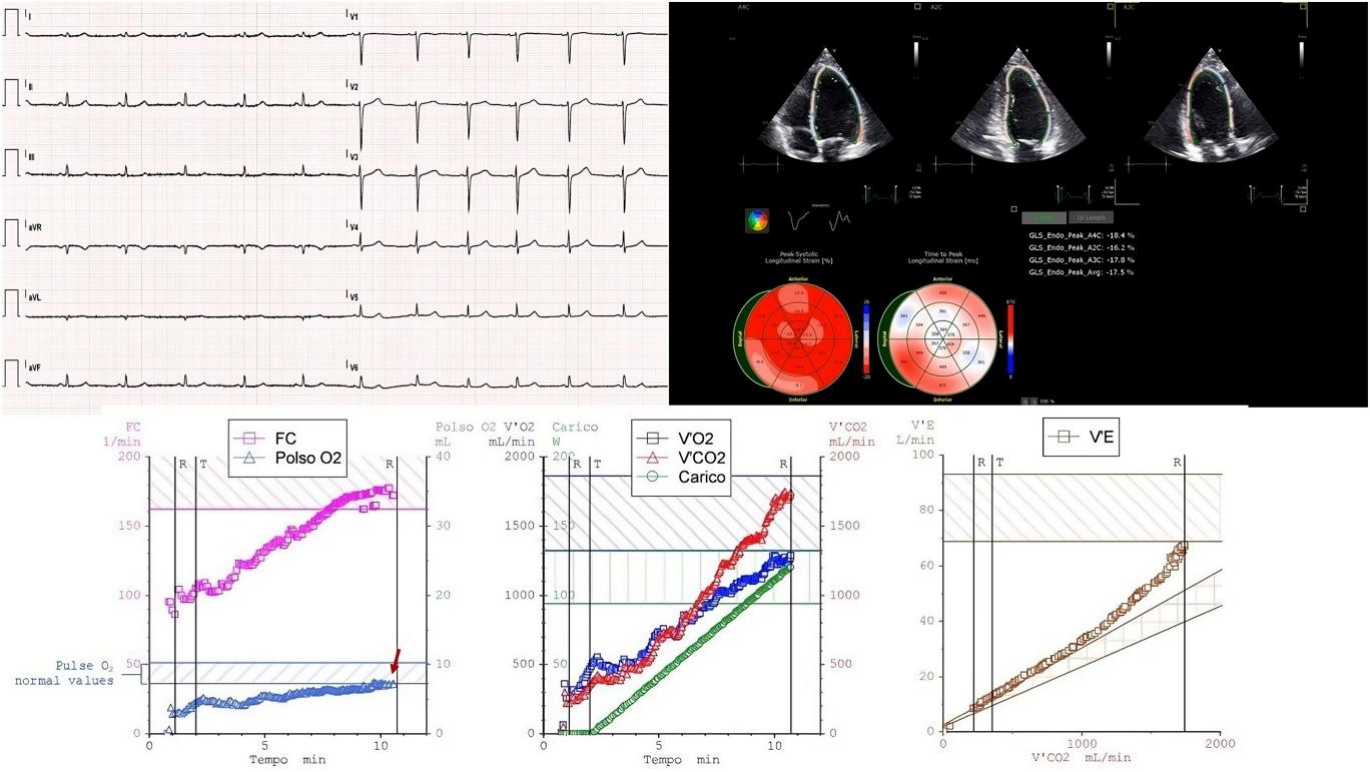
Relative 2, carrier of a pathogenetic titin truncating variant. (*Upper-left side*) Electrocardiogram shows a sinus rhythm at 70 b.p.m, normal atrioventricular and interventricular conduction, normal repolarization, of note, low voltages of the QRS in the limb leads. (*Upper-right side*) The speckle tracking analysis shows a left ventricular dysfunction with global longitudinal strain of −18%. (*Lower side*) Cardiopulmonary exercise testing graphs: left panel shows the O_2_ pulse and heart rate behaviour; the arrow indicates the reduced O_2_ pulse values; central panel shows VO_2_ and VCO_2_ kinetic and workload; right panel shows VE/VCO_2_ slope of 30.9.

At first evaluation, the female was asymptomatic (no palpitations, syncope, or presyncope reported), with no clinical signs of HF. Electrocardiogram (*Figure 1*) showed a normal atrioventricular and interventricular conduction and a normal depolarization and repolarization pattern. A notable non-specific finding was the reduction in amplitude of the QRS voltages in the limb leads, although it did not meet the criterion for low QRS voltages (amplitude < 5 mm in every limb lead). The echocardiogram was normal, including LV diameters and systolic function [LV end-diastolic diameter (LVEDD) 41 mm, LV end-diastolic volume (LVEDV) 58 mL, LVEF 55%, and LV-GLS −18%). A significant burden of ventricular premature beats (VPBs) of more than 500 was recorded on a 24-h Holter ECG. Cardiac magnetic resonance was refused due to claustrophobia.

To further evaluate symptoms status and hemodynamic stability, a CPET was performed (*Figure 1*): the test was maximal [respiratory exchange ratio (RER) = 1.36] and showed a peak oxygen uptake (VO_2_) of 27 mL/kg/min, which was at the lower limit of normal (80% of predicted VO_2_ peak). A reduced peak oxygen pulse (peak O_2_ pulse) was observed (7.2 mL/min, 83% of predicted), with a flat curve and no evident down-sloping. The chronotropic response was normal [max heart rate (HR) 172/min, 95% of predicted]. The anaerobic threshold was found to be within the normal range. The minute ventilation to carbon dioxide production slope (VE/VCO_2_ slope) was at the upper limit value (30.9), and ventilatory parameters were within normal limits. Electrocardiographic monitoring revealed a single isolated ventricular extrasystole (left bundle branch block pattern, inferior axis) during the effort phase.

In conclusion, the low peak VO_2_ (although still within the normal range in absolute terms) and the reduced oxygen pulse may reflect a reduced cardiac performance compared with normal, regardless of the subject's training status. Indeed, in relation to these CPET subtle functional abnormalities, even in the absence of a diagnosis of DCM or NDLVC, the subject will be monitored on an annual basis.

## Relative 4: filamin C

A 34-year-old female was seen for the first time in the context of family screening. Her father (the proband) was diagnosed with DCM caused by an autosomal dominant heterozygous P variant in filamin C (*FLNC*) gene (NM_001458.5: c.7251+1G>A). Genetic testing showed the presence of the same heterozygous variant in the daughter.

Family history did not include any cases of sudden cardiac death. The patient reported having played volleyball at a competitive level for 10 years until 10 years ago, maintaining an active lifestyle since then. She reported being an active smoker (10 cigarettes per day) since the age of 15.

**Figure 2:**
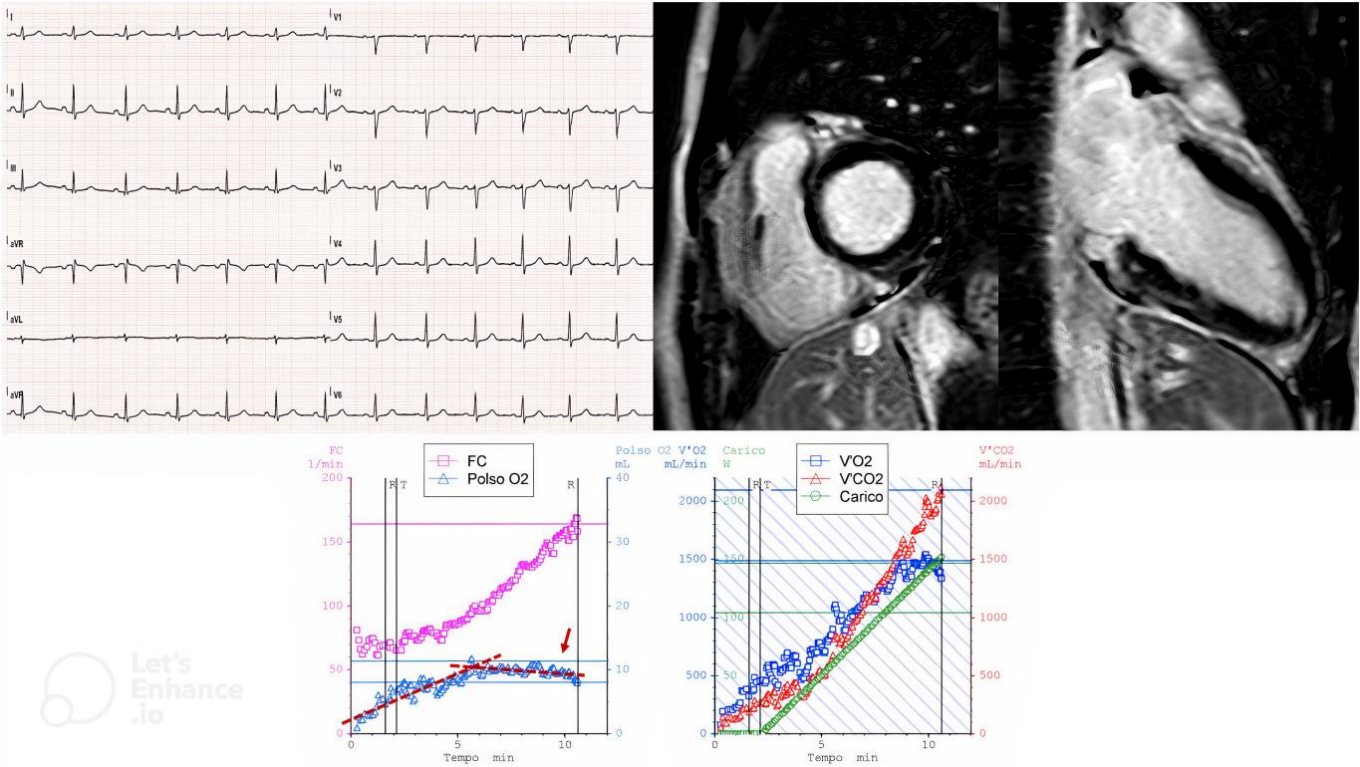
Relative 4, carrier of a pathogenetic variant of filamin C. (*Upper left side*) Electrocardiogram shows a sinus rhythm, normal atrioventricular and interventricular conduction, normal depolarization, and repolarization patterns. (*Upper-right side*) Cardiac magnetic resonance reveals the presence of a subepicardial late gadolinium enhancement in the basal inferior segment. (*Lower side*) Cardiopulmonary exercise testing graphs: left panel shows the heart rate behaviour and the O_2_ pulse early flattening and a slight down-sloping at the peak of the exercise, as indicated by the arrow; right panel shows VO_2_ and VCO_2_ kinetic and workload.

On the first visit, the patient was not taking any medication and reported sporadic episodes of palpitations. The ECG revealed a sinus rhythm, a normal atrioventricular and interventricular conduction, and a normal depolarization and repolarization pattern (*Figure 2*). Echocardiography revealed no cardiac abnormalities (LVEDD 51 mm, LVEDV 87 mL, LVEF 56%, and LV-GLS −20%). The 24-h ECG Holter monitoring did not record any significant ventricular arrhythmias.

Cardiopulmonary exercise testing was performed to evaluate the patient’s functional capacity and to exclude the presence of inducible arrhythmias (*Figure 2*). The exam was conducted on the same day of the visit, in the absence of drug therapy.

Cardiopulmonary exercise testing was maximal (RER = 1.54) and demonstrated a peak VO_2_ of 24 mL/kg/min, which was slightly reduced in comparison with the predicted value (76% of predicted VO_2_ peak) in the absence of physical deconditioning. Furthermore, a mild reduction of the peak O_2_ pulse (82% of predicted) with an early flattening and a slight down-sloping at the peak of exercise was observed, and the chronotropic response was normal (max HR 168/min, 91% of predicted).

The anaerobic threshold was within normal range. Pulmonary vascular impairment was absent, as evidenced by a normal VE/VCO_2_ slope of 25.6. Normal ventilatory parameters were observed. During electrocardiographic monitoring, isolated monomorphic VPBs (right bundle branch block pattern and superior axis) became more frequent with exercise once the HR exceeded 120 b.p.m. and regressed completely during the recovery phase.

The evaluation was integrated by a CMR (*Figure 2*), which revealed normal dimensions of the LV, a LVEF at the lower limit of normal (LVEF 55%), and the presence of a subepicardial late gadolinium enhancement (LGE) in the basal inferior segment (a potential origin site of VPBs).

Due to the familial pathogenetic and arrhythmogenic FLNC variant, characterized by a high burden of VPBs and evidence of fibrotic involvement of the LV, cardioactive therapy with a low dose of beta-blocker (bisoprolol) has been initiated. The therapy was effective in reducing arrhythmic burden on Holter ECG at 2 months and a clinical stability at 6 months. Here, CPET was effective in detecting a low cardiac performance with a pathological behaviour of O_2_-pulse.

## Discussion

Dilated cardiomyopathy and NDLVC, caused by genetic mutation, often show variable intrafamilial penetrance and phenotypic variability. Additionally, environmental ‘triggers’ (such as hypertension, obesity, chemotherapy, peri- and postpartum status, and strenuous physical exertion) may influence manifestation. These factors complicate family screening, particularly when limited to ECG and echocardiography, which are sometimes insufficient to provide a precise and accurate evaluation.

In recent years, the clinical definition of phenotype-negative among genotype-positive relatives has been reconsidered. Historically, negative ECG and echo were together sufficient to assign a relative as unaffected.^3,12^ However, incorporation of more advanced imaging techniques, such as CMR, in accordance with recent European guidelines, demonstrated that some relatives previously classified as genotype-positive/phenotype-negative had already developed cardiac fibrosis.^2^ Left ventricular dysfunction with global longitudinal strain has also proven effective in detecting subtle left ventricular dysfunction, even where normal LVEF is reported,^4–6^ while Holter ECG monitoring has revealed frequent VPBs and episodes of non-sustained ventricular tachycardia. The finding of exercise-induced arrhythmias carries a prognostic significance for future cardiomyopathy development.^13^

These findings reinforce an increasingly clear understanding in the field of genetic cardiomyopathies: the LVEF alone is insufficient not only as a prognostic tool but also for diagnostic purposes, and further functional tests may be needed.

Cardiopulmonary exercise testing is not routinely used to study genotype-positive/phenotype-negative relatives, but it has a clinically relevant role in the context of manifest cardiomyopathies. Specifically, CPET has been shown to predict long-term HF events and, consequently, disease progression.^9^ It is utilized in clinical practice for the titration of HF therapy and to determine appropriate follow-up intervals.

Our data set revealed relatives with normal LV dimensions and functions who were carriers of P/LP familial variants that showed abnormalities upon additional testing: four relatives exhibited abnormalities on ECG, two on echocardiography (LV-GLS < −18%), four on CMR with non-ischemic LGE, and three on Holter ECG with ventricular arrhythmia. Furthermore, two relatives demonstrated subtle functional abnormalities on CPET, suggesting a more advanced myocardial involvement. Specifically, a slight reduction or stabilization of the O_2_ pulse at maximal HR in these subjects may reflect an early myocardial dysfunction, as it is accompanied by other abnormalities.^14^

In this regard, family screening, particularly for genotype-positive individuals, should be multiparametric in the definition of phenotype. Despite a normal resting heart function, individuals may still have an underlying condition that makes the heart muscle less efficient during exercise compared with a genotype-negative control.

In this context, the CPET could assume a functional role in terms of cardiac performance definition; however, it should be noted that peak VO_2_ measurements can be misleading, particularly in individuals with normal or near-normal hearts. According to Fick's law, exercise performance depends on several factors, including cardiac output and O_2_ extraction by muscles. Sedentary individuals with normal hearts may have lower peak VO_2_ than physically active patients with HF, leading to significant overlap between these groups.^15,16^

However, as demonstrated by the two cases here, CPET has the potential to serve as a valuable and informative tool, including in circumstances where CMR is not readily available or when the examination is not feasible.

The follow-up interval for genotype-positive/phenotype-negative relatives suggested by the European guidelines is quite broad, ranging from 1 to 3 years.^2^ Given the role of CPET in manifest cardiomyopathies in predicting events and disease progression,^9^ an abnormal CPET in these two subjects could suggest that the clinician opts for a shorter follow-up interval, such as 1 year or even less.

Finally, as there are no studies indicating that early initiation of therapy in family members can slow or alter the potential future manifestation of the disease, subtle functional abnormalities, such as those identified by CPET, might one day support the introduction of cardioprotective therapy, considering these subjects were already affected by an early stage of myocardial disease.

In our case series, the detection of subtle functional abnormalities on CPET, together with other findings, guided specific clinical decisions. For Relative 2, we chose to undertake a shorter follow-up at 1 year, accompanied by a full clinical and instrumental re-evaluation (not yet conducted at the time of writing). For Relative 4, we initiated beta-blocker therapy to reduce the arrhythmic burden, which could itself be a contributing factor to the subtle functional abnormalities detected on CPET and, in the long term, to LV systolic function impairment.^17^

In conclusion, in our case series, the CPET contributed to the diagnostic assessment of genotype-positive relatives with a normal LVEF, aiding in defining the extent of overt disease manifestation and supporting clinical decision-making relating to follow-up intervals and therapy initiation.

Further analyses with extended follow-up data are needed to explore the role of CPET in predicting the development of overt cardiomyopathy or cardiac events in genotype-positive relatives who are phenotype-negative or exhibit only minor signs of the disease.

## Lead author biography


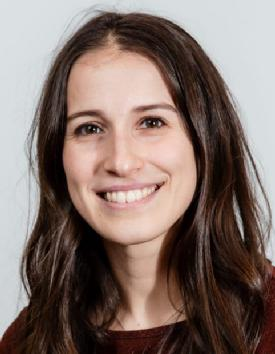


Eva Del Mestre is a cardiology resident at the Cardiovascular Department, University of Trieste (Italy). The Cardiology Unit of Trieste is a reference centre for the diagnosis and treatment of cardiomyopathies and member of the ERN GUARD—Heart. Her interests focus on cardiomyopathies and in particular on family screening for cardiomyopathies.


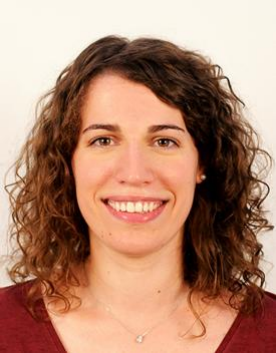


Teresa Maria Capovilla is a cardiology resident at the Cardiovascular Department, University of Trieste (Italy). The Cardiology Unit of Trieste is a reference centre for the diagnosis and treatment of cardiomyopathies and member of the ERN GUARD—Heart. She has a special interest in heart failure, cardiomyopathies, and cardiopulmonary exercise testing.

## Supplementary Material

ytaf162_Supplementary_Data

## Data Availability

The data underlying this article will be shared on reasonable request to the corresponding author.

## Notes

**Consent:** The authors confirm that written consent for submission and publication of this case report including the images and associated text has been obtained from all the patients in line with COPE guidance.

**Funding:** None declared.
