# Supplementary material for: The contribution of cardiopulmonary exercise testing in the familial screening for dilated and non-dilated left ventricular cardiomyopathies: case series
Source: Eur Heart J Case Rep. 2025 Apr 8;9(4):ytaf162. doi: 10.1093/ehjcr/ytaf162 (PMC12038895; doi:10.1093/ehjcr/ytaf162)
Supplement: ytaf162_Supplementary_Data [file ytaf162_supplementary_data.docx]

**Supplementary Material**

**Cardiopulmonary exercise test protocol**

In all patients, a CPET using an individualised cycle-ergometer ramp protocol, as previously described, was performed to assess functional capacity.(1s) In the absence of clinical events, CPET was symptom-limited and self-interrupted by patients regardless of the peak respiratory exchange ratio (RER) achieved.

A “breath-by-breath” analysis of respiratory gases and ventilation was performed and all measurements were calculated and reported in accordance with the methodology described by Mezzani.(2s) PeakVO2 was defined as the highest measured VO2 rate during the final 30 seconds of the CPET. The Wasserman equation, adjusted for age and sex, was used to calculate the percentage of predicted peakVO2 (%VO2 peak).(3s) The anaerobic threshold (AT) was calculated by the V-Slope graphic analysis of carbon dioxide production (VCO2) and VO2, therefore confirmed by examining ventilatory equivalents and end-tidal pressures of CO2 and O2. Premature AT was defined by values <40% of the predicted % peakVO2.(4s) Peak O2-pulse was determined as peakVO2/peakHR. The Minute ventilation-to-carbon dioxide output (VE/VCO2) slope was calculated as the linear relationship between VE and VCO2 from 1 minute after the onset of loaded exercise until the end of the isocapnic buffering period as previously outlined.

Predicted maximal HR was defined using the Fox formula (220 – age). The VyntusTM CPX Metabolic Cart (Vyaire Medical®) was the equipment used in this study.

Electrocardiography, blood pressure and peripheral oxygen saturation were documented before, during and after the exercise.

**References**

1s. Agostoni P, Dumitrescu D. How to perform and report a cardiopulmonary exercise test in patients with chronic heart failure. Int J Cardiol. 2019 Aug 1;288:107-113.

2s. Mezzani A. Cardiopulmonary Exercise Testing: Basics of Methodology and Measurements. Ann Am Thorac Soc. 2017 Jul;14(Supplement_1):S3-S11.

3s. Wasserman K. Principles of Exercise Testing and Interpretation. Wolters Kluwer Health/Lippincott Williams & Wilkins 2012, ed. 5th edition. 2012.

4s. Salvioni E, Mapelli M, Bonomi A, Magrì D, Piepoli M, Frigerio M, et al. Pick Your Threshold: A Comparison Among Different Methods of Anaerobic Threshold Evaluation in Heart Failure Prognostic Assessment. Chest. 2022 Nov;162(5):1106-1115.
